# Towards an Objective Measure of Mindfulness: Replicating and Extending the Features of the Breath-Counting Task

**DOI:** 10.1007/s12671-017-0880-1

**Published:** 2018-01-22

**Authors:** Kian F. Wong, Stijn A. A. Massar, Michael W. L. Chee, Julian Lim

**Affiliations:** Center for Cognitive Neuroscience, Neuroscience and Behavioral Disorders Department, Duke-NUS Medical School, 8 College Road, #02-21, Singapore, 169857 Singapore

**Keywords:** Trait mindfulness, Objective measurement, Breath counting, Attentional lapses, Mind-wandering

## Abstract

Despite calls for objective measures of mindfulness to be adopted in the field, such practices have not yet become established. Recently, a breath-counting task (BCT) was proposed as a reliable and valid candidate for such an instrument. In this study, we show that the psychometric properties of the BCT are reproducible in a sample of 127 Asian undergraduates. Specifically, accuracy on the BCT was associated with everyday lapses and sustained attention, and weakly associated with subjectively measured mindfulness. BCT metrics also showed good test-retest reliability. Extending the use of the paradigm, we further found that two different types of task errors—miscounts and resets—were correlated with different aspects of cognition. Miscounts, or errors made without awareness, were associated with attentional lapses, whereas resets, or self-caught errors, were associated with mind-wandering. The BCT may be a suitable candidate for the standardized measurement of mindfulness that could be used in addition to mindfulness questionnaires.

## Introduction

Since the introduction of the mindfulness-based stress reduction program developed by Kabat-Zinn (1991), there has been substantial interest in training and increasing mindfulness to improve health and well-being. Indeed, quantitative reviews have shown that mindfulness training has beneficial effects on a wide suite of health outcomes, including reducing stress and anxiety, strengthening emotional regulation, and improving cognition in general (Eberth and Sedlmeier 2012; Sedlmeier et al. 2012).

A less studied observation in the field is that trait-like individual differences in mindfulness exist even in the absence of any formal mindfulness training (Brown and Ryan 2003). These may owe to personality differences that arise from early experience, or the influence of genetic polymorphisms (Tang et al. 2015). Natural variations in trait mindfulness predict cognitive performance (Fountain-Zaragoza et al. 2016), everyday driving ability (Burdett et al. 2016), and psychological health (Bodenlos et al. 2015; Tamagawa et al. 2013). These benefits may be mediated by the increased tendency of those low on trait mindfulness to mind wander more (Mrazek et al. 2012; Wang et al. 2017). In support of this, high trait mind-wandering is also associated with negative outcomes such as unhappiness (Killingsworth and Gilbert 2010) and poorer cognitive performance (Franklin et al. 2016; Smallwood et al. 2004).

At present, trait mindfulness is most commonly measured using subjective self-report (Bergomi et al. 2013; Sauer et al. 2013), with the Mindful Awareness and Attention Scale (MAAS; Brown and Ryan 2003) being one of the most frequently cited of these instruments. Subjective scales commonly attempt to survey at least one of the two major components of mindfulness, which are defined as (1) observing and attending to present-moment thoughts and experiences with (2) an attitude of non-judgment, curiosity, and acceptance, with the MAAS primarily capturing elements of the first.

Scales such as the MAAS have been a useful assay of dispositional mindfulness; however, relying solely on them has several drawbacks. As with all self-report instruments, participant responses may be biased by factors such as experimenter demand or social desirability. Specific to this field, asking an individual to introspect on their level of mindfulness has an element of circularity—non-mindful individuals may not even be aware of their lack of mindfulness, or may not understand what a question about mindfulness is asking of them. As such, it would be useful to have an objective measure of mindfulness to complement the data obtained from subjective scales, particularly in populations where the reliability and validity of mindfulness questionnaires might be called into doubt, for example, participants with no prior exposure to mindfulness training, or those with difficulty comprehending mindfulness scales due to a low education level or cognitive impairment.

Recently, Levinson et al. (2014) proposed a breath-counting test (BCT) as just such an objective measure. This paradigm has good face validity, as it is similar to the mindful-breathing exercises taught in most mindfulness-based training courses, and in Vipassana meditation. Importantly, it also has convergent validity with meta-awareness and non-attachment, and discriminant validity with other cognitive constructs such as sustained attention and working memory. These authors also demonstrated that breath-counting accuracy increased with mindfulness, but not working memory training. While good breath-counting ability may not directly test all aspects of mindfulness, it still appears to adequately measure both attention-related and non-attention-related facets of the construct.

Levinson et al. (2014) used total accuracy rate as the main dependent variable in their analyses and did not investigate their properties of the types of errors in detail. Two types of error may arise: resets (termed as “self-caught miscount” by Levinson et al.) and miscounts (uncaught errors). While Levinson et al. (2014) do distinguish between these errors in their report, they do not investigate their properties in detail.

Our aim in the current experiment was to replicate several of the key findings of Levinson et al. (2014), as well as to further explore the two different types of errors that can be committed on the task with the following a priori hypotheses. First, we predicted that miscounts and resets would represent two uncorrelated but reliable classes of error. Second, we predicted that miscounts would be detrimental to sustained attention, representing task disengagement that occurs outside of awareness, while resets would be correlated with mind-wandering. We also conducted exploratory analyses on the association between the breath-counting error types and subjective reports of trait mindfulness and everyday cognitive lapses.

## Method

### Participants

Students from a local university in Singapore volunteered to participate (*N* = 127; mean age (sd) = 23.4 (2.8), female = 74), by responding to an advertisement posted on the student portal and were compensated ten Singapore dollars each for a 1-h session. Two data sets were incomplete due to errors in stimulus presentation and were excluded from analysis. Of the original batch of participants, *N* = 39 (mean age (sd) = 23.3 (2.85), 26 females) returned for a second session, based on their performance during the first session, and were compensated 25 Singapore dollars for the 2-h session which included a 30-min fMRI scan. All testing sessions occurred between 1 pm and 5 pm to control for circadian confounds. The study was approved by the National University of Singapore Institutional Review Board and was conducted in accordance with the ethical standards of the 1964 Helsinki declaration and its later amendments. All participants provided written informed consent.

### Procedure

During the first session, participants performed a 20-min breath-counting task (BCT; Levinson et al. 2014), and a 20-min version of the psychomotor vigilance test (PVT; Lim and Dinges 2008), and completed the Mindfulness Attention Awareness Scale (MAAS; Brown and Ryan 2003), Cognitive Failures Questionnaire (CFQ; Broadbent et al. 1982), and Mind-Wandering Questionnaire (MWQ; Mrazek et al. 2013).

During the second testing session, participants performed a second trial of the 20-min BCT (Levinson et al. 2014). After this, they underwent a 30-min fMRI scan (data not reported here). All stimuli and questionnaires were presented using Psychtoolbox (Brainard 1997).

### Measures

#### Breath-Counting Task

During the BCT, participants were seated comfortably in front of an LCD monitor with both hands resting on a standard QWERTY keyboard. They were given the following instructions, which were taken verbatim from Levinson et al. (personal communication, 7 September, 2016) with the additional instruction to not count the breaths using their fingers.

In this task, we would like you to be aware of your breath. Please be aware of the movement of breath in and out in the space below your nose and above your upper lip. There’s no need to control the breath. Just breathe normally.

At some point, you may notice your attention has wandered from the breath. That’s okay. Just gently place it back on the breath.

To help attention stay with the breath, you’ll use a small part of your attention to silently count breaths from 1 to 9, again and again. An in and out breath together makes one count. Say the count softly in your mind so it only gets a little attention while most of the attention is on feeling the breath. Please press the <Left Arrow> using your index finger with breaths 1-8, and the <Right Arrow> using your 4th finger with breath 9. This means you’ll be pressing a button with each breath.

If you find that you have forgotten the count, just press the <SPACE> with your left index finger. You can restart the count at 1 with the next breath. Do not count the breaths using your fingers but only in your head.

We suggest you sit in an upright, relaxed posture that feels comfortable. Please keep your eyes at least partly open and resting on the screen during the experiment. The task will last about 20 minutes.

Breathing rate was recorded at 32 Hz using a portable recording device (SOMNOtouch RESP, SOMNOmedics GmbH, Germany) with an effort band that was applied around the abdomen of the participant. Synchronization was done manually and the recording was started when the participants triggered a 3-s countdown to begin the task. Once the task started, the screen went blank. Each BCT lasted 20 min. Breathing data was extracted using DOMINOlight software (SOMNOmedics GmbH, Germany) and exported for analysis.

#### Psychomotor Vigilance Task

The PVT is a demanding assay of sustained attention that has been deployed in operational settings (Basner and Dinges 2011; Lim and Dinges 2008). Participants were instructed to maintain their attention to a rectangular box on the screen and to respond with the spacebar using their dominant hand the moment a millisecond time counter appeared. They were instructed to respond as quickly as possible to each trial without anticipating the appearance of the counter. A 20-min PVT was used instead of the standard 10-min version to increase the number of lapses (response times > 500 ms) committed, and exaggerate inter-individual variability in performance. Median response speed (RS, or reciprocal reaction time) was also calculated. RS and lapses are the two most sensitive outcome measures on the PVT (Basner and Dinges 2011).

#### Questionnaires

Three questionnaires were administered in session 1 of the testing. Participants completed the MAAS (Brown and Ryan 2003), which consists of 15 questions and is used to measure trait levels of mindful attention. The Cognitive Failures Questionnaire (CFQ; Broadbent et al. 1982) is a 25-item questionnaire which measures everyday failures in perception, memory, and motor functions. The last questionnaire administered was the Mind-Wandering Questionnaire (MWQ), which consists of five questions measuring trait levels of task-unrelated thoughts with each item being rated on a 1–6 scale. Questionnaires were scored using the templates described in their respective original papers.

### Data Analyses

A small number of participants (*N* = 7) were not compliant to the breath-counting instructions, and performed at zero, or near-zero accuracy in the task (0–7%). These participants were removed from the sample before further analysis. Three participants were outliers in their number of resets (14, 26, and 32) that drove correlations with several outcome variables; these too were removed from the sample. One of these participants had both a high number of resets and poor accuracy. Thus, the final sample used for analysis consisted of *N* = 116 participants (mean age (sd) = 22.70 (2.77), female = 69).

Breath-counting data were broken down into count cycles that terminated with either the right arrow key press or spacebar key press. A count cycle containing eight left arrow key presses followed by one right arrow key press was considered a correct cycle. Any count cycle that ended with a right arrow key press without eight left arrow key presses preceding it was considered a *miscount* cycle while cycles that ended with a spacebar key press was considered a *reset* cycle. Overall accuracy was calculated as the number of correct count cycles over the total number of count cycles.

In addition to these metrics, we removed instances of what we term *singles*, which we defined as two right arrow key presses in a row. We reasoned that such trials are highly likely to represent mechanical errors rather than genuine miscounts. The mean and standard deviation of singles in our sample was 1.04 (2.07).

We note that there were a small number of key presses that occurred too close together to be physiologically plausible (mean = 0.37, sd = 1.43). Although these were also likely to represent motor errors, in these instances, we could not tell what post-hoc decision the participant made (to include or exclude the slip as part of a cycle count). As such, we opted to leave these trials in the analysis for simplicity.

Pearson correlations were used to examine the relation between BCT performance, PVT performance, and subjective measurements of mindfulness. All analyses were performed with IBM Statistical Package for the Social Sciences (SPSS) 24.0.0.0 for Mac.

## Results

### Breath-Counting Performance

To ensure that participants were pressing a button on every inhalation, we correlated the total number of button presses per participant with their total breath count as measured by breathing rate. These variables were nearly perfectly correlated (*r* = .96).

Mean (sd) accuracy on the BCT was 72.05% (17.62). Even after exclusions, there were substantial inter-individual differences in BCT performance, with accuracy ranging from 20 to 100%. Importantly, accuracy was not correlated with the total number of breath cycles across participants (*r* = .09, *p* = .32).

**Fig. 1:**
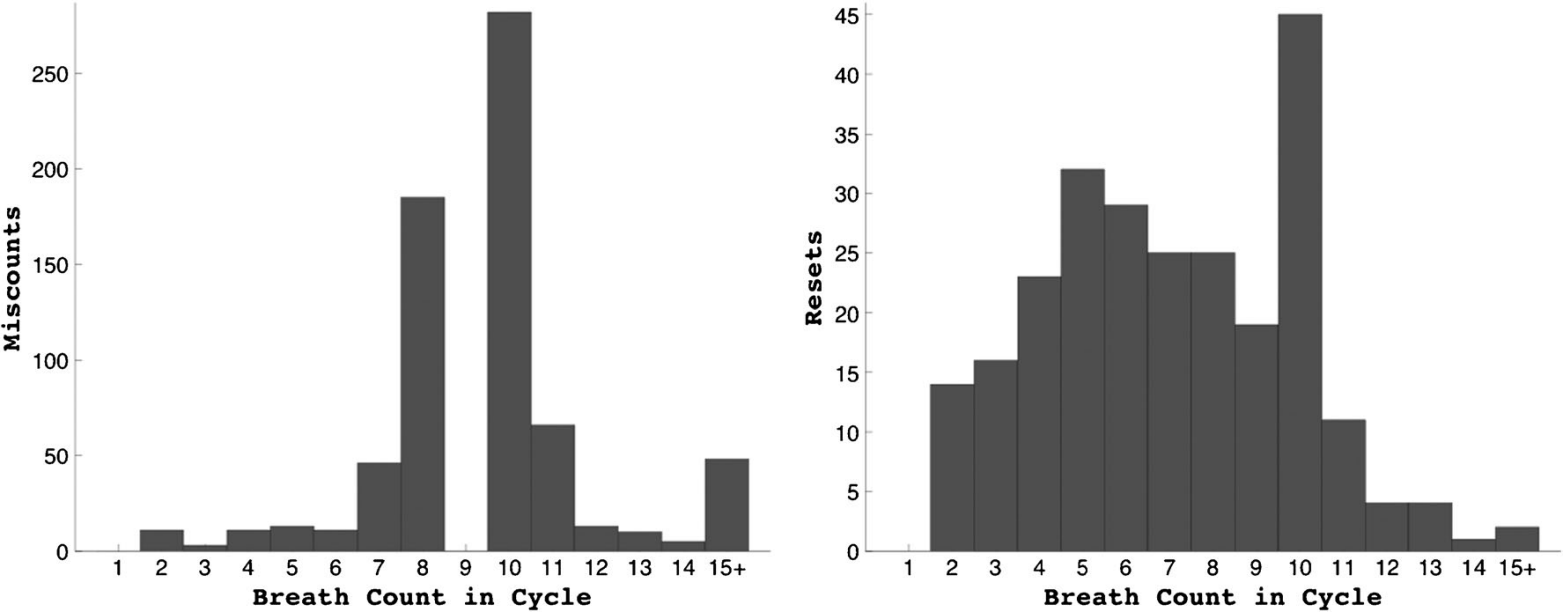
Histograms charting the total number of miscounts and resets for each breath in the cycle. Note that bin 1 is empty because these trials were considered to be inadvertent errors (singles) and not entered into analysis

There was large inter-individual variability in the type of error committed, with the proportion of miscounts/resets ranging from 0 to 100%. Miscounts were more common than resets (76.99 vs. 23.01%). Figure 1 depicts the distribution of miscounts and resets within the breath cycle; as expected, miscounts cluster strongly around the ninth breath, whereas resets are more equally distributed within the breath cycle. We normalized the number of miscounts and resets by the total number of breath cycles to obtain miscount and reset rates before subjecting these variables to further analysis.

We asked whether miscounts and resets represent independent types of errors by correlating the rates of these two variables. There was no significant correlation between miscounts and resets (*r* = .043, *p* = .79).

### Time-on-Task Effects

On the suggestion of a reviewer, we tested for and documented time-on-task effects on the BCT by comparing performance in the first and second half of the task. Participants performed significantly worse in the second half of the task compared to the first on all three metrics (accuracy: t_115_ = 7.16, *p* < 10^−11^; miscounts: t_115_ = − 2.70, *p* = .008; resets: t_115_ = − 2.68, *p* = .009).

### Reliability of Breath-Counting Accuracy

To assess the reliability of the BCT accuracy measures, a subset of good (top tertile; *N* = 21, mean (sd) accuracy = 90.2% (5.5) and poor (bottom tertile; *N* = 17, mean (sd) accuracy = 56.1 (9.2)%)) performers were recalled for a second testing session (mean (sd) of test-retest interval = 54.1 (49.9) days)) during which they also underwent fMRI scanning (not reported in this manuscript). We performed two-way mixed inter-class correlations (ICC) on BCT accuracy, miscount rate, and reset rate across these two sessions. All these variables showed fair to good reliability over time (accuracy: ICC = .48, *p* = .001; miscounts: ICC = .47, *p* = .001; resets: ICC = .72, *p* < .0001). Miscounts and resets remained uncorrelated in the second testing session (*r* = − .005, *p* = .97).

We note that recalling extreme groups of participants may cause ICC to be underestimated due to regression to the mean. Accordingly, our reliability of 0.48 for overall accuracy is lower than the ICC of 0.6 reported by Levinson et al. (2014), and the value reported in that original paper may be a better estimate of BCT reliability.

In addition to test-retest reliability, we tested for split-half reliability (first vs. second half) of the BCT. We found moderate reliability on all three of the metrics tested (accuracy = .57, *p* < 10^−11^; miscounts = .55, *p* < 10^−10^; resets = .33, *p* = .0003).

### PVT Performance

Median (sd) response speed (reciprocal reaction time) on the 20-min PVT was 3.20 (0.36). The mean (sd) number of lapses (RT > 500 ms) was 8.37 (10.04). Lapses were not normally distributed (Shapiro-Wilk = .76, *p* < .001); however, we still report parametric statistics for this variable as our sample is large.

### Relationship Between Breath-Counting and Sustained Attention

**Fig. 2:**
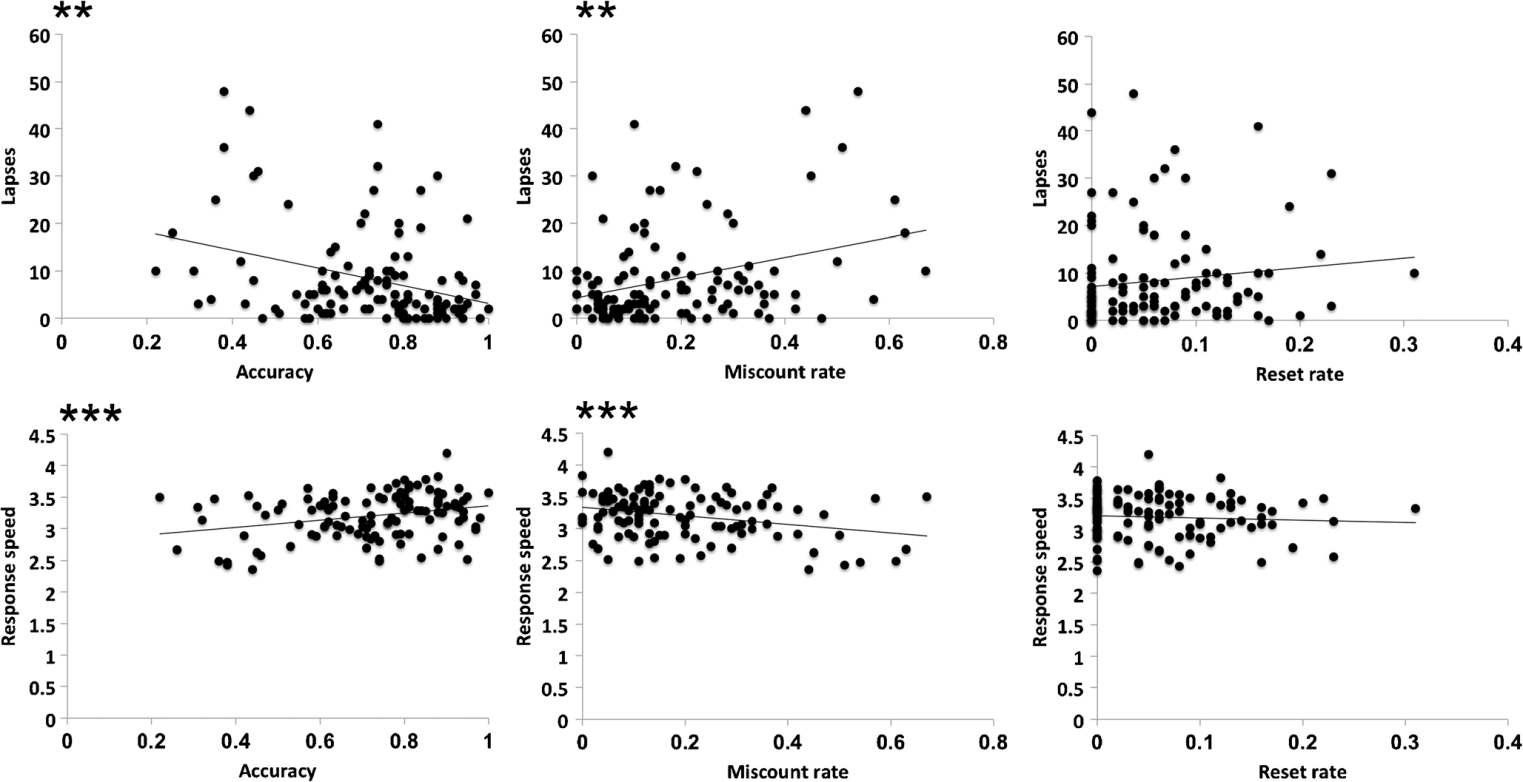
Key outcome variables on the psychomotor vigilance task (response speed and lapses (RT > 500 ms)) were significantly correlated with overall accuracy and miscounts on the breath-counting task. In contrast, resets were not significantly correlated with either of these measures of sustained attention. **p < .01, ***p < .001

We performed correlation analysis between BCT variables (accuracy, miscounts, and resets) and the two outcome variables from the PVT (RRT and lapses). Results are summarized in Table 1 and Fig. 2. We found significant correlations between accuracy and both PVT outcome variables. Miscounts were also significantly correlated with RRT and lapses. Resets did not correlate with either of the PVT outcomes.

**Table 1:** Pearson’s correlation values (*r*) between the breath-counting task and psychomotor vigilance test variables

		Breath-counting task					
		Accuracy		Miscount rate		Reset rate	
		*r*	*p*	*r*	*p*	*r*	*p*
Psychomotor vigilance test	Response speed	.282**	.002	− .290**	.002	− .065	.48
	Lapses	− .328***	< .001	.323***	< .001	.129	.17

**p < .01

***p < .001

### Association with Subjective Scales

**Fig. 3:**
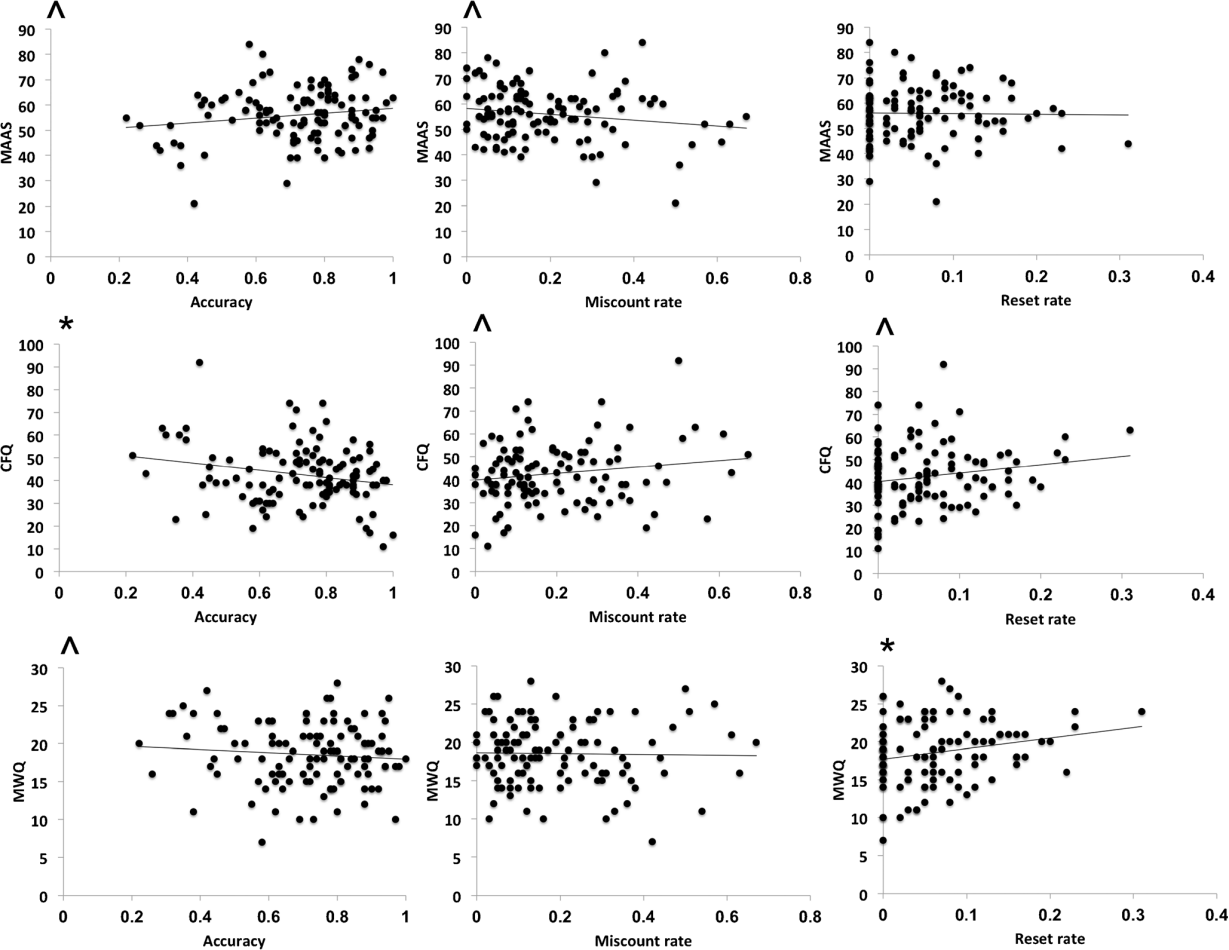
(top) Breath-counting accuracy and miscounts are marginally correlated with trait mindfulness measured using the Mindful Awareness and Attention Scale (MAAS), (middle) breath-counting miscounts and resets are and significantly correlated (*p* = .03) with everyday lapses of attention measured by the Cognitive Failures Questionnaire (CFQ). ^*p* < .1, *p < .05

We next correlated the three BCT variables with the scores on the MAAS, the MWQ, and the CFQ, noting that the scores on the three questionnaires were highly inter-correlated (MAAS/CFQ: *r* = − .67, *p* < .001; MAAS/MWQ: *r* = − .46, *p* < .001; CFQ/MWQ: *r* = − .43, *p* < .001). Results are summarized in Table 2 and Fig. 3. In brief, overall accuracy was associated at least at the trend level with all three scales. In addition, in the current dataset, we observed a dissociation whereby miscounts tended to be correlated with the attentional aspects of mindfulness (as measured by the MAAS and CFQ), whereas resets were associated with mind-wandering (as measured by the MWQ).

**Table 2:** Pearson’s correlation values (*r*) between breath-counting task and subjective measures

	Breath-counting task					
	Accuracy		Miscount rate		Reset rate	
	*r*	*p*	*r*	*p*	*r*	*p*
MAAS	.164^	.08	− .169^	.07	.129	.17
CFQ	− .213*	.02	.165^	.08	.181^	.05
MWQ	− .093^	.32	− .016	.86	.219*	.02

*MAAS*, Mindful Awareness and Attention Scale; *CFQ*, Cognitive Failures Questionnaire; *MWQ*, Mind-Wandering Questionnaire

^*p* < .1

*p < .05

We used Fisher r-to-z transformation to compare our MAAS/BCT accuracy correlation with the result obtained by Levinson et al. (2014) (*r* = .20, *p* = .05), and found no significant difference between these two correlations (z = 0.28, *p* = .78). Thus, although the correlation falls below the threshold of statistical significance here, they still lie within the confidence interval of the original finding.

## Discussion

We conducted a study to replicate and extend the results of Levinson et al. (2014), who validated a breath-counting task as an objective measure of trait mindfulness. In line with the original study, we found good test-retest reliability of performance on the BCT, as well as correlations with sustained attention and self-reported mindfulness. Additionally, we report a dissociation between two types of errors that can be committed on the BCT: miscounts and resets. Although the rates of committing both types of errors are stable within individuals across time, our data suggest that miscounts are associated directly with lapses in attention, while resets are associated with self-reported mind wandering.

Currently, trait mindfulness is most commonly measured via the use of self-report instruments. Self-reported mindfulness increases with mindfulness training and meditation experience (Baer et al. 2008; Brown and Ryan 2003; Chambers et al. 2007), and these increases correlate with desirable outcomes such as decreased burnout and stress (Roeser et al. 2013), lower depression and anxiety scores (Desrosiers et al. 2013), better executive functioning (Black et al. 2011), and more optimized patterns of brain functional connectivity (Mooneyham et al. 2017). In summary, subjective scales have sufficient reliability and validity to measure mindfulness under many circumstances.

However, there are also limitations to the exclusive use of self-report instruments. At present, a diversity of scales exists, each measuring slightly different facets of mindfulness, making it challenging to compare findings across different studies. Moreover, none of the current available scales comprehensively measures all aspects of mindfulness (Bergomi et al. 2013). Self-report measures are also vulnerable to various forms of response bias (Grossman 2008, 2011). For instance, social desirability has been shown to correlate with responding on the MAAS (Brown and Ryan 2003). Cognitive dissonance may also play a role in significant changes due to mindfulness training; the significant investment of time and effort into a program may induce individuals to believe they are more mindful, even in the absence of objective evidence that this is the case (Grossman 2008). Furthermore, the exposure to mindfulness training may itself prompt people to endorse higher levels of mindfulness in subjective report due to increasing familiarity with the concepts and lexicon used by practitioners.

Alternatives to subjective measurement have been proposed. These include qualitative assessment (e.g., using interview data), assessment by others, or neuropsychological approaches; Grossman 2008, 2011; Sauer et al. 2013). However, none of these is yet widely used in the field. The BCT is one of the first objective tests of mindfulness to be developed, and is in our opinion a promising candidate that could be used to supplement self-reported mindfulness measures. Our current data support this opinion by replicating some key reliability and validity metrics of the test, as well as demonstrating its applicability in a non-Western setting.

To summarize the findings we replicated the following: BCT performance showed good test-retest reliability, showed a trend correlation with subjectively measured mindfulness (MAAS scores), and a significant correlation with performance on a test of sustained attention. While Levinson et al. (2014) reported correlations with the Sustained Attention to Response Test (Robertson et al. 1997), here, we find associations with key outcome variables on the Psychomotor Vigilance Task, a sensitive assay of vigilance that is used in operational settings (Basner and Dinges 2011; Lim and Dinges 2008).

In line with the original validation study, we found that BCT performance explained ~ 10% of the variance in PVT performance. This supports the discriminant validity of the BCT —the test is not simply measuring the ability to sustain attention. We note that the resemblance of the BCT to a sustained or selective attention test may call its construct validity as a measurement of mindfulness into question. Levinson et al. (2014) have gone some way towards mitigating this concern: in one of their experiments, they show that BCT performance correlates with non-attachment (the ability to resist a rewarding distractor). Moreover, the task instructions do not emphasize accuracy and in fact explicitly tell participants to place only “a small part of their attention” on the count. Consequently, mean BCT accuracy in this sample (72.1%) is relatively low compared to what is typically seen in sustained/selective attention tests (80–90%; e.g., Dillard et al. 2014; Robertson et al. 1997) suggesting that our participants were not focusing their attention narrowly on the breath to “do well.” Having said this, we do not have any direct evidence of BCT performance relating to non-judgment or non-attachment in the current study, and more experiments addressing this relationship would be valuable.

In the current experiment, we extend the analysis of the BCT by distinguishing between miscounts and resets. While these two error types are uncorrelated, they each also have reasonable-to-good test-retest reliability (miscounts: ICC = 0.48; resets: ICC = 0.72), suggesting that they might be capturing separate trait-like aspects of mindfulness.

To further investigate this, we correlated these error types with our questionnaire data. We found that miscounts were marginally associated with scores on the MAAS, suggesting that they relate to attentional lapses, or periods of task disengagement without awareness. In support of this, miscounts but not resets were correlated with lapses on the PVT, which are a robust indicator of momentary attentional failures (Lim and Dinges 2008). While neither miscounts nor overall accuracy were significantly correlated with the MAAS at α = 0.05, our estimate (*r* = .18) did not differ significantly from Levinson et al. (2014) (*r* = .20). The combined evidence thus supports a weak correlation between BCT performance and MAAS.

In contrast, resets were correlated with MWQ scores, indicating that these errors may relate more closely to mind wandering. This is presumably mind-wandering that occurs with meta-awareness, since participants must be aware that they are mind-wandering in order to endorse high scores on the MWQ. As resets occur only when a participant realizes that their attention is no longer on counting the breath, it is reasonable to expect this variable to be correlated with the amount of mind wandering the individual is aware of engaging in during everyday life. Interestingly, we found no correlation between miscounts and MWQ scores, suggesting that miscounts are not strongly related to the interference of task-unrelated thoughts (with meta-awareness), but are more likely to be the result of momentary failures of attention/working memory or mind-wandering without meta-awareness. However, this is an open hypothesis that remains to be tested.

While mindfulness/meta-awareness is negatively correlated with both mind-wandering and failures of attention, these two outcomes may not result from identical underlying processes (Mrazek et al. 2013). This distinction has been clearly drawn in the literature on mind wandering, which can occur with or without meta-awareness (Smallwood and Schooler 2006). We speculate that miscounts and resets on the BCT may be useful covert metrics that disentangle the consequences of a lack of mindfulness—mind-wandering with meta-awareness, which could be benign or even intentional (Seli et al. 2016) and lapses in attention, which may occur outside of meta-awareness, and have more serious real-world consequences (Dinges 1995). We note, however, that both miscounts and resets are associated with scores on the CFQ, suggesting that both of these errors might ultimately play a role in everyday mistakes that occur due to a lack of mindfulness.

Finally, we propose that the BCT may also be useful for cross-cultural research. The semantic understanding of certain mindfulness scale items can differ between populations (Grossman 2008), and this issue is compounded if there is a need to compare scale scores across languages. The BCT circumvents this problem, as it is unlikely that comprehension or semantic interpretation would result in differences across cultures when performing this test. Furthermore, the simplicity of the BCT instructions indicates its utility for populations where language comprehension is impaired. Future studies could profitably investigate whether the reliability and validity of the BCT are superior to self-report measures in patients with cognitive impairment, poorer language abilities, or who are simply not accustomed to providing data via questionnaires.

Ultimately, the greatest benefit of using a standardized objective measure of mindfulness is that it permits robust comparison of results across laboratories, facilitating the comparison of intervention methods, and easing quantitative synthesis or meta-analysis. The positive features of the BCT make it a promising instrument that could potentially fill that role. With the ever-increasing number of trials being conducted to examine the effects of mindfulness interventions, the use of a task such as the BCT may be critical to harmonize and make comparable results from across the field.
